# PD27 Budget Impact Analysis Of The High-Dose Trivalent Influenza Vaccine For The Brazilian Unified Health System

**DOI:** 10.1017/S0266462325101621

**Published:** 2025-12-29

**Authors:** Márcia Gisele Costa, Kátia Senna, Bernardo Tura, Carlos Magliano, Ricardo Fernandes, Marisa Santos

## Abstract

**Introduction:**

Influenza is an acute infectious disease that is highly transmissible and affects the respiratory system. It impacts the health system and society by reducing a patient’s productivity and quality of life. The population group most susceptible to complications and death is adults older than 65 years and children younger than five years.

**Methods:**

The analysis was performed from the perspective of the Unified Health System (SUS) as the funder. The eligible population was estimated based on epidemiological data from population projections by the Brazilian Institute of Geography and Statistics for a five-year time horizon. The assumption of 90 percent vaccination coverage was adopted as market share. Analyses were performed by scenarios that varied market share and added costs with clinical benefits. Costs were estimated on a national basis, considering the price of the technology, inputs, and waste. Hospitalization costs and their confidence intervals were obtained from the SUS Hospitalization Authorization database.

**Results:**

The cost of vaccination with the high-dose trivalent influenza vaccine was estimated at USD119,637,999.04 in the first year and USD657,405,529.68 over a five-year time horizon. The increase in the budget was USD104,034,983.12 in the first year and USD571,747,563.22 over five years. The alternative scenario one was estimated considering vaccination coverage in 2023 of 63.3 percent, obtaining an incremental impact of USD72,824,488.19 in the first year and USD400,167,351.63 in five years. Alternative scenario two considered the reduction in hospitalizations and found an incremental impact of USD103,908,194.74 in the first year and USD570,970,948.59 in five years.

**Conclusions:**

The budgetary impact was estimated at over USD571 million in five years. In absolute numbers, the reduction in hospitalizations due to influenza (0.4%) and pneumonia (0.3%) was not very significant, which generated a reduction in the incremental budgetary impact, in relation to the main scenario of the analysis, of 0.02 percent in the first year and 0.13 percent in five years.

